# Development and external validation of a FDG PET-based radiomics model predicting occult lymph node metastasis in non-small cell lung cancer patients

**DOI:** 10.1007/s00259-025-07740-y

**Published:** 2026-01-16

**Authors:** Vincent Bourbonne, P. Lovinfosse, M. Geier, R. Le Pennec, R. Abgral, K. Pluchon, JN Choplain, B. Duysinx, F. Lallemand, Arnaud Uguen, R. Hustinx, R. Magwenzi, M. Hatt, O. Pradier, F. Lucia

**Affiliations:** 1 University of Western Brittany, INSERM, UMR 1101, LaTIM, Brest, France; 2 Radiation Oncology Department, University Hospital of Brest, Brest, France; 3 Division of Nuclear Medicine and Oncological Imaging, University Hospital of Liège, Liège, Belgium; 4 GIGA-Laboratory of Experimental Nuclear Medicine, University of Liège, Liège, Belgium; 5 University of Western Brittany, INSERM, UMR 1304, GETBO, Brest, France; 6 Medical Oncology Department, University Hospital of Brest, Brest, France; 7 Nuclear Medicine Department, University Hospital of Brest, Brest, France; 8 Thoracic Surgery Department, University Hospital of Brest, Brest, France; 9 Pneumology Department, University Hospital of Liège, Liège, Belgium; 10 Radiation Oncology Department, University Hospital of Liège, Liège, Belgium; 11 Pathology Department, University Hospital of Brest, Brest, France; 12 University of Western Brittany, INSERM, UMR 1227, LBAI, Brest, France

**Keywords:** Occult lymph node metastasis, Radiomics, PET-CT, Surgery, Stereotactic radiotherapy

## Abstract

**Purpose/Objective(s):**

Accurate detection of occult lymph node metastasis (OLNM) in patients with localized non-small cell lung cancer (NSCLC) remains a clinical challenge. This study aimed to develop and validate a radiomics-based predictive model for OLNM.

**Materials/Methods:**

A radiomics model (Model_PET_) and a model (Model_Combined_) combining radiomics and clinical features were developed using a retrospective monocentric cohort of localized NSCLC patients treated with surgery (Cohort A) and tested on an external cohort (Cohort B) of 112 localized NSCLC patients also treated with surgery (publicly available Radiogenomics cohort). The model was further assessed in an independent cohort of 488 patients with localized NSCLC who underwent definitive stereotactic body radiotherapy (SBRT) (Cohort C) using regional relapse free survival (RRFS) as a surrogate for OLNM. Radiomic features were extracted from pre-treatment FDG PET and combined to predict OLNM using a multilayer perceptron approach.

**Results:**

In the training cohort, the Model_PET_ and Model_Combined_ achieved AUCs of 0.92/0.99 and balanced accuracies (Bacc) of 80.0%/85.3%, respectively. In the Cohort B, the Model_PET_ and Model_Combined_ resulted in AUCs of 0.73/0.67 and Baccs of 71.2%/51.7%, respectively. In the Cohort C, the predicted OLNM risk based on Model_PET_ was significantly associated with worse RFFS (HR 1.60 95% CI 1.03–2.48, *p* = 0.04). The Model_Combined_ was not associated with survival outcomes (*p* > 0.05).

**Conclusion:**

This study presents a radiomics-based predictive model for OLNM in localized NSCLC, validated across several retrospective independent cohorts. Subject to a prospective evaluation, the model could be used to refine clinical decision-making.

**Supplementary Information:**

The online version contains supplementary material available at 10.1007/s00259-025-07740-y.

## Introduction

Lymph node involvement (LNI) is a key prognostic feature in all cancer staging. LNI significantly impacts survival as well as therapeutic management. For non-small cell lung cancer (NSCLC), the AJCC 9th edition classifies disease with LNI as stage IIA or upper cases [1]. For IA, IB and node-negative (N0) IIA stages, surgery alone is the gold standard option [2]. When not feasible, stereotactic radiotherapy (SBRT) is the main option, achieving 5y local control rates of 89.6% but an increased rate of mediastinal relapse [3, 4]. Indeed, up to approximately 20% of patients will experience mediastinal relapse after SBRT (vs. 10% in case of lobectomy) [5].

Fluorodeoxyglucose positron emission tomography/computed tomography ([^18^F]FDG PET/CT) has transformed pre-operative and pre-RT imaging workup of lung cancer [6]. For mediastinal node staging, previous studies report negative predictive values above 90%, largely outperforming computed tomography [7–9]. Occult lymph node metastasis (OLNM) is a major issue. In surgical candidates, OLNM can be diagnosed through systematic lymph node dissection. In contrast, patients treated with SBRT do not undergo nodal sampling, making OLNM a plausible explanation for their higher rates of regional relapse.

The SEISMIC study evaluated the benefit of systematic endobronchial ultrasound-guided transbronchial needle aspiration (EBUS), resulting in a 37% discrepancy between the PET-CT and the EBUS. The OLNM rate was 12% [10]. Access to EBUS remains difficult, limiting the feasibility of systematic invasive nodal staging.

Several imaging-models, especially radiomics and deep learning models, have been developed. Models based on CT and PET/CT have shown promising internal performance [11–15] with AUC values reaching 0.84 for CT-based models and 0.90 for PET/CT-based models [16]. These studies share key limitations, being the histology specificity (focus on adenocarcinoma, excluding other histologies) [9, 17], the lack of external validation and the paucity of transferability to other treatment strategies, such as SBRT. To our knowledge, the study by Zhong et al. remains the largest to include multicenter cohorts but did not evaluate the transferability to a cohort treated by SBRT [18].

In the present study, we developed and externally validated a radiomics-model for the prediction of OLNM in patients treated with surgery or SBRT for a stage I-II NSCLC.

## Materials and methods

### Population

This multicentric study focused on localized NSCLC patients treated either with surgery or SBRT, with a curative intent. The cohorts A and C included only stage I-II NSCLC while the cohort B focused on stage I-III NSCLC. Other inclusion criteria included age > 18 years, available [^18^F]FDG PET/CT performed before treatment not showing any sign of advanced disease, normal brain imaging (CT or MRI). Histology confirmation was not mandatory. When not performed, decision for treatment had to rely on international guidelines and multidisciplinary board [2].

This study was a multicentric and international study, involving several cohorts. Two institutions took part in the project: The University Hospital of Brest and the University Hospital of Liège. Patients from the University Hospital of Brest were treated using surgery or SBRT while patients the University Hospital of Liège were managed using SBRT only. Patients from the Radiogenomics cohort (surgical cohort from the Stanford Cardiothoracic Surgery Department, Palo Alto, California, USA and from the Veteran Affairs Medical Center, Los Angeles, USA, both available on The Cancer Imaging Archive : TCIA) were also included [19, 20].

The surgical cohorts from the University Hospital of Brest and from Radiogenomics were defined as Cohorts A and B, respectively. The SBRT cohorts from the University Hospital of Brest and from the University Hospital of Liège were merged as the Cohort C.

The study design was accepted by the Institutional Review Boards of Liège University Hospital (2022/285) and Brest University Hospital (29BRC25.0162). The Institutional Review Board of Liège University Hospital exempted the need for informed consent for the patients from CHU Liège. For the cohort from the Brest University Hospital, all patients consented to the use of their data.

The study was conducted in accordance with the Declaration of Helsinki.

## PET/CT imaging

In the cohort A, PET/CTs were performed with 3 types of scanners: from 2010 to 2012 on an analog Gemini GXLi (Philips© Healthcare, Netherlands) system, from 2012 to 2018 on an analog Biograph-mCT (Siemens©, Erlangen, Germany) system and from 2019 to 2024 on a digital Vision 600 (Siemens©, Erlangen, Germany) system. In the cohort B, PET/CTs were performed with 4 types of scanners: a GE Discovery STE, a GE Discovery 690 and a GE LightSpeed VCT and a Siemens scanner for which relevant DICOM headers (0008,1090: Model name) were deleted for de-identification when submitted as a TCIA collection.

In the cohort C, PET/CTs were performed with 3 types of scanners. For patients from University Hospital of Liège, studies were acquired with cross-calibrated Philips Gemini TF or BB (Philips Healthcare) while for patients from University Hospital of Brest, studies were acquired with the same scanners as in the Cohort A.

## Surgery planning and lymph node dissection

Patients from the Cohort A were operated on by experienced thoracic surgeons in the thoracic surgery department of the Brest University Hospital, between 2010 and 2024.

Patients from the Cohort B were operated on by experienced thoracic surgeons either at the Stanford Thoracic surgery department or the Veteran Affairs Thoracic surgery department, both in Los Angeles (CA, USA), between 1990 and 1996.

Surgeries were performed in accordance with the national and international guidelines in effect at the time of their execution.

Lymph node status was determined based on the pathology report and classified as N0 (no LNI) versus N + in case of LNI. N1 and N2 status were differentiated for sub-analyses.

## SBRT planning and delivery

At the University Hospital of Liège, studies were acquired with a Philips Brilliance Big Bore CT (Philips Healthcare). At the University Hospital of Brest, studies were performed with a Siemens, Somatom (Siemens Healthcare, Malvern, PA, USA). No contrast-enhancing agent was used.

SBRT regimens were chosen based on the tumour localization (peripheral, central or ultra-central), tumour volume and patients’ comorbidities according to the international guidelines for lung SBRT [21]. Only fractionated protocols were used. At the University Hospital of Liège, SBRT was delivered using a Cyberknife platform (Accuray Incorporated, Sunnyvale, CA, USA) between 2010 and 2020 while at the University Hospital of Brest, SBRT was delivered using a Truebeam Novalis STX (Varian Medical Systems, Palo Alto, CA, USA) equipped with a standard Millennium MultiLeaf (MLC) collimator with 120 leaves, between 2016 and 2020.

## Extraction of radiomics

PET-CT were retrospectively retrieved and semi-automatically segmented using the PET-Edge tool (MIM v 7.1.4, MIM Software, Cleveland, OH, USA). Segmentations were performed by three different readers, a single reader being responsible of a single cohort (A: R.M, B: V.B and C: F.L). All segmentations were reviewed by two experienced radiation oncologists (F.L and V.B). Radiomics features were extracted using the PyRadiomics v3.0.1 python package, in accordance with the IBSI (imaging biomarker standardization initiative) guidelines [22].

Radiomic features were extracted after 1 × 1 × 1 mm^3^ spatial resampling of all PET images using a cubic spline interpolation. CT images were not considered because of the heterogeneity of CT acquisition especially regarding contrast enhancement and the known impact on radiomics features [23]. For the calculation of the texture matrix-based features, image intensities were discretized using fixed bin width (FBW of 0.1). As a result, 75 (without filtering) and 744 (after wavelet filtering) radiomic features were available for each patient. Given the multicentric design and the temporal heterogeneity of our database, normalization and post-hoc harmonization were performed following the methodologies by Fortin et al. [24, 25] and Da-Ano et al. [26]. Normalization was performed separately on each cohort, before post-hoc harmonization. The NeuroCombat a posteriori statistical harmonization method was performed on the training cohort. Harmonization parameters were then transferred to Cohorts B and C following the methodology by Da-Ano et al..

### Feature set reduction and model Building

The following methodology was used in several previous studies and has shown promising results [27–30].

Selection of features of interest was performed using the Mann–Whitney test after harmonization and normalization. Only statistically associated features in regard to the outcome were retained (*p* < 0.05). Inter-features correlation was assessed with the Spearman correlation coefficient, keeping only the most significant feature in case of a Spearman coefficient ≥ 0.7.

The model was built to predict OLNM using a decremental neural network approach (Multilayer Perceptron, IPSS Modeler, v18.0, IBM, NY, USA). Initial tuning parameters were defined as follows: the softmax activation function, an initial lambda of 5.10^− 7^, an initial sigma of 5.10^− 5^, an interval center of 0 with an interval offset of +/- 0.5. A single hidden layer was used. OLNM was defined as pathologically proven N + at surgery despite cN0 on PET/CT. To account for the low OLNM risk and the inherent risk of overfitting, the development cohort was randomly partitioned as 60% of the cohort was used for building the model and 40% for internal validation. A 10-cross fold validation approach was used, relying on 10 separate models being built changing the random seed accordingly. An ensemble model compiled the 10 models’ prediction by averaging each model’s prediction. At each step, the feature with the lowest importance was deleted and the models retrained. The Youden index (YI) calculated on the Cohort A was used for the selection of both the best model and the probability threshold. The model was evaluated on both the training and testing cohorts using the receiver operative characteristics including the Balanced accuracy (Bacc) and the F1 score. For completeness, a second model (Model_Combined_) combining clinical (tumour stage based on AJCC 9th edition [1], age, tumour histology, gender) and radiomics data was built following the same methodology.

Feature set reduction and model building were performed on the cross-validation folds of Cohort A. The internal validation fold remaining untouched. The cohort B was used as the testing cohort while evaluation for regional relapse-free survival prediction was performed on the cohort C.

An exploratory analysis was performed for the prediction of the N2 status. Using the same model, the probability threshold was refined on the training cohort to maximize the YI using the N2 status as the classification variable.

Regarding the Cohort C (SBRT cohort), we used Kaplan-Meier survival analysis to estimate regional relapse-free survival (RRFS) and overall survival (OS). RRFS was defined as the delay between SBRT completion and regional relapse, defined as the appearance of suspicious (PET-positive nodal uptake with size/avidity increase confirmed in multidisciplinary tumour board) or histologically proven mediastinal lymph node (or last known visit). OS was defined as the delay between SBRT completion and death (or last known visit). Impact of the model and each probability threshold (depending on the endpoint: LNI and N2) was assessed on 5y RRFS and OS using the restricted mean survival approach [31]. Kaplan Meier curves were also compared using the log rank test. Decision curves were plotted for each Model. Statistical analyses were performed using SPSS Statistics v27.0 and MedCalc v20.104.

The Radiomics Quality Score was calculated [32]. The study was conducted in accordance with the TRIPOD-AI guidelines [33].

## Results

### Population characteristics

**Fig. 1:**
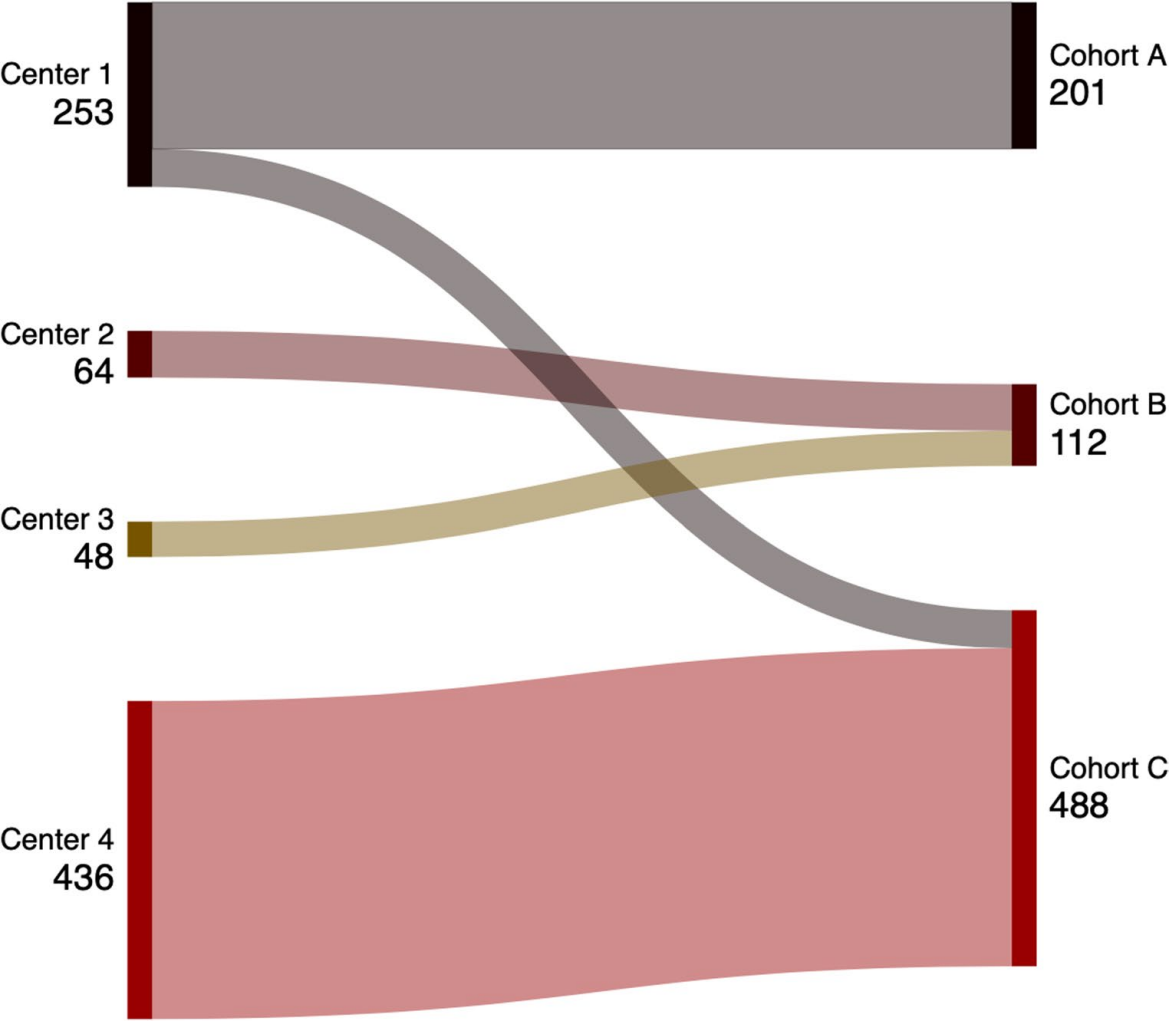
Distribution of patients according to the center and the cohort

The study included three distinct cohorts of patients with localized NSCLC, each with specific characteristics. Cohort A consisted of 201 patients, with a median age of 65.5 years and a predominance of adenocarcinoma histology (87.6%). Cohort B included 112 patients from the publicly available Radiogenomics dataset, with a slightly older median age of 69.0 years and a higher proportion of squamous cell carcinoma (23.2%) compared to Cohort A. The OLNM were relatively similar in the two cohorts: 13.0% and 10.7% in Cohorts A and B, respectively. Lastly, Cohort C was the largest, comprising 488 patients. This group had the oldest median age (72.6 years) and a more diverse histological profile (43.2% adenocarcinoma, 34.8% squamous cell carcinoma). Histology was not available for 87/488 patients (17.8%). The large majority of patients from Cohort C (436/488) were from the University Hospital of Liège. The main patients’ characteristics are summarized in Table 1 while the link between centers and cohorts is explicated with Fig. 1.

**Table 1:** Characteristics of the population

Cohort			A (*n* = 201)	B (*n* = 112)	C (*n* = 488)
Age (Median, IQR			65.5 (59.5–71.0)	69.0 (63.0–76.0)	72.6 (65.9–80.0)
Gender: F/M (nb, %)			62 (30.9)/139 (69.1)	28 (25.0)/84 (75.0)	180 (36.9)/308 (63.1)
Histology	Adenocarcinoma		176 (87.6)	83 (74.1)	211 (43.2)
	SCC		21 (10.4)	26 (23.2)	170 (34.8)
	NSCLC NOS		3 (1.5)	3 (2.7)	20 (4.1)
	Not available		1 (0.5)	0 (0.0)	87 (17.8)
Tumor stage/AJCC stage (nb/%)	T1a – IA1		44 (21.9)	30 (26.8)	67 (13.7)
	T1b – IA2		43 (21.4)		231 (47.3)
	T1c – IA3		17 (8.5)	21 (18.8)	120 (24.6)
	T2a – IB		82 (40.8)	32 (28.6)	53 (10.9)
	T2b – IIA		14 (7.0)	8 (7.1)	17 (3.5)
	T3 – IIB		1 (0.5)	15 (13.3)	0 (0.0)
	T4 - IIIA		0 (0.0)	6 (5.4)	0 (0.0)
Pathological LNI status	N0		175 (87.0)	100 (89.3)	NA
	N+	N1	17 (8.5)	4 (3.6)	NA
		N2	9 (4.5)	8 (7.1)	NA

Abbreviations: *IQR* Interquartile range, *F* Female, *M* Male, *SCC* Squamous Cell Carcinoma, *NSCLC* Non-small cell lung carcinoma, *NOS* Not otherwise specified, *AJCC* American Joint Committee on Cancer, *LNI* Lymph Node Involvement

### Development of the model

#### Results of the model in the training cohort

Thirteen radiomics features were retained after the feature set reduction step (Supplementary Table 1). When combined, the 13 radiomics models achieved relatively high performances, with YI ranging from 0.37 up to 0.60 (Supplementary Fig. 1). The highest performing ensemble model for Model_PET_ combined 11 different radiomics features through 10 models, each sub-model including 4 radiomics features each. Overall, importance of radiomics features ranged from 2.2% up to 16.4% and resulted in an AUC of 0.92 (95% CI 0.85–0.96, *p* < 0.0001), F1 score 0.57. Full details of Model_PET_ are provided in the Supplementary Table 2.

The highest performing ensemble for Model_Combined_ combined 17 features among which all 4 clinical features were retained. Each sub-model included 15 features. Age was the most important feature accounting for 17.5% of the prediction (Supplementary Table 2). It reached an AUC of 0.99 (95% CI 0.95–1.00.95.00, *p* < 0.0001), F1 score 0.92.

### Results of the model in the internal validation cohort

In the internal validation cohort, the Model_PET_ resulted in an AUC of 0.70 (95% CI 0.59–0.79, *p* = 0.02), F1 score 0.42 while the Model_Combined_ resulted in an AUC of 0.74 (95% CI 0.63–0.82, *p* = 0.004), F1 score 0.55.

### Clinical benefit

**Fig. 2:**
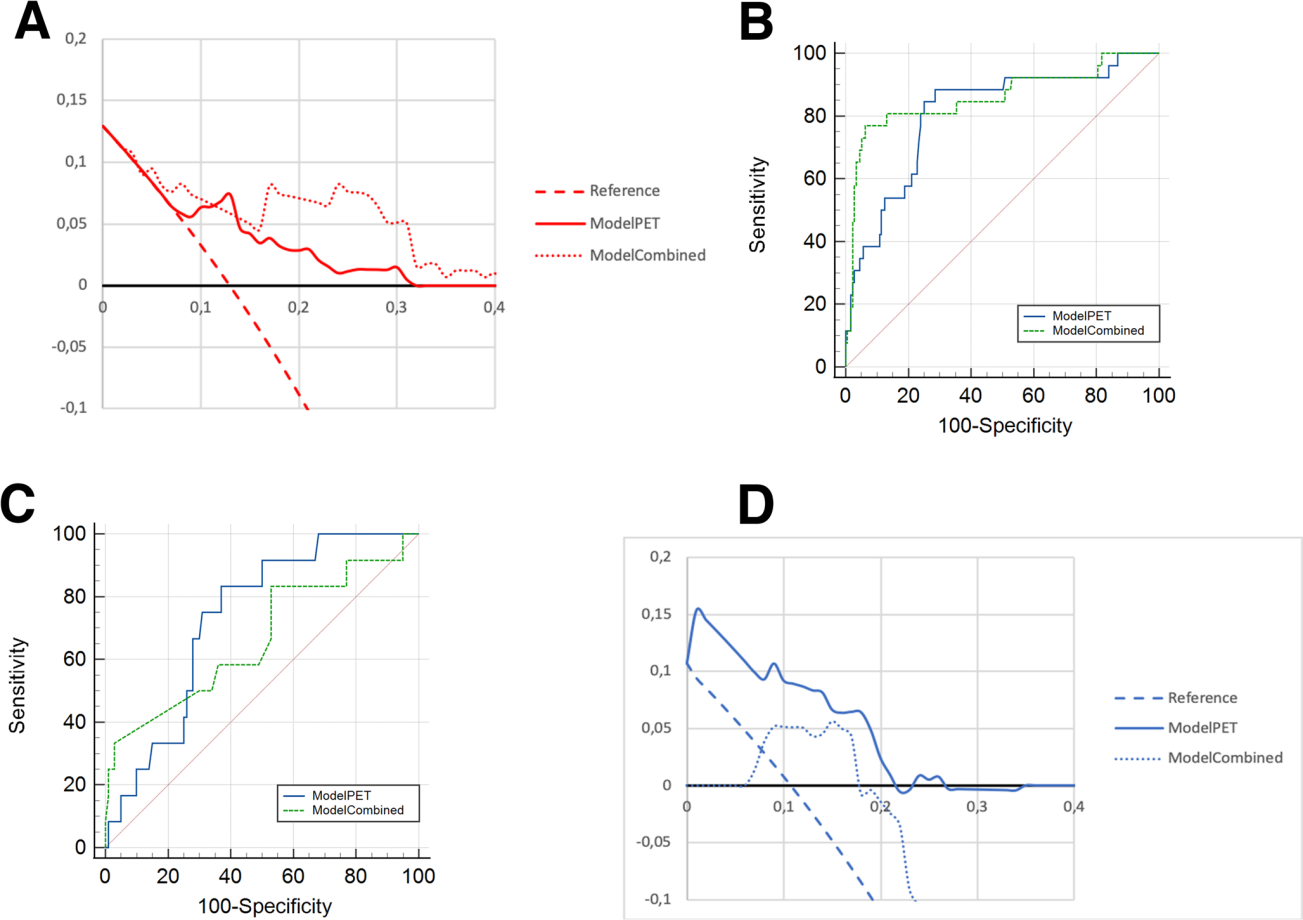
Decision curve analysis and ROC curves for the ModelPET and the ModelCombined in the Cohorts A and B

By maximizing the YI, the probability threshold for the Model_PET_ was set at 12.5%, resulting in a Bacc of 80.0% (sensitivity (Se) of 88.5%, a specificity (Sp) of 71.4%) and a Negative Predictive Value (NPV) of 97.7% (95% CI 93.5–99.2) for the Cohort A. The decision curve analysis showed the benefit of the Model_PET_ against PET only (Fig. 2a) while the ROC curve for the Cohort A is provided as Fig. 2b. Regarding the Model_Combined_, the probability threshold set at 24% was associated with a Bacc of 85.3% (Se 76.9%, Sp 93.7%).

### Results of the model in the surgical testing cohort

In the Cohort B, the Model_PET_ achieved a YI of 0.46, an AUC of 0.73 (95% CI 0.64–0.81, *p* = 0.0002), F1 score 0.35. Applying the 12.5% probability threshold resulted in a Bacc of 71.2%, Se of 83.3% and Sp of 59.0%. The NPV of the Model_PET_ was 96.7% (95% CI 89.2–99.1) compared to 89.3% for the PET alone. The Model_Combined_ resulted in an AUC of 0.67 (95% CI 0.57–0.75, *p* = 0.08) and a Bacc of 51.7%, with a Se of 8.3%. ROC curves are provided as Fig. 2c. Patients with OLNM were significantly older in Cohort B than in Cohort A with a median age of 69.0 years (Interquartile range – IQR: 63.0–76.0) vs. 65.5 years (IQR 59.4–71.0), *p* < 0.001. Decision curve analysis confirmed the clinical utility of the Model_PET_ (Fig. 2d): the net benefit of Model_PET_ exceeded PET-alone for threshold probabilities 5–25%.

Performance of the different models according to the cohort are summarized in Table 2 while Supplementary Fig. 1 provides the distribution of YI for both models.

**Table 2:** Results of the Model_PET_ and the Model_Combined_ in the training and testing cohorts

Model	Cohort	AUC (95% CI)	F1 score	Bacc (%)	Se (%, 95% CI)	Sp (%, 95% CI)	TN	FN	FP	TP	PPV (%, 95% CI)	NPV (%, 95% CI)
Model_PET_	Cohort A	0.81 (0.75–0.86)	0.48	80.0	88.5 (69.9–97.6)	71.4 (64.1–78.0)	125	3	50	23	31.5 (26.0–37.7.0.7)	97.7 (93.5–99.2)
	Cohort B	0.73 (0.64–0.81)	0.35	71.2	83.3 (51.6–97.9)	59.0 (48.7–68.7)	59	2	41	10	19.6 (14.7–25.6)	96.7 (89.2–99.1)
Model_Combined_	Cohort A	0.86 (0.80–0.90)	0.70	85.3	76.9 (56.4–91.0)	93.7 (89.0–96.8.0.8)	164	6	11	20	64.5 (49.7–76.9)	96.5 (93.1–98.2)
	Cohort B	0.67 (0.57–0.75)	0.42	51.7	8.3 (0.2–38.5)	95.0 (88.7–98.4)	95	11	5	1	16.7 (2.5–61.1)	89.6 (87.9–91.2)

Abbreviations: *AUC* Area Under the Curve, *CI* Confidence Interval, *Bacc* Balanced accuracy, *Se* Sensitivity, *Sp* Specificity, *TN* True Negative, *Nb* Number, *FN* False Negative, *FP* False Positive, *TP* True Positive

Calibration was moderate and varied greatly depending on the model and the cohort as shown in Supplementary Figs. 2a-b and Supplementary Table 3. External calibration for the Model_PET_ was acceptable (intercept 0.404, slope 1.487).

### Evaluation of the model in the SBRT application cohort

**Fig. 3:**
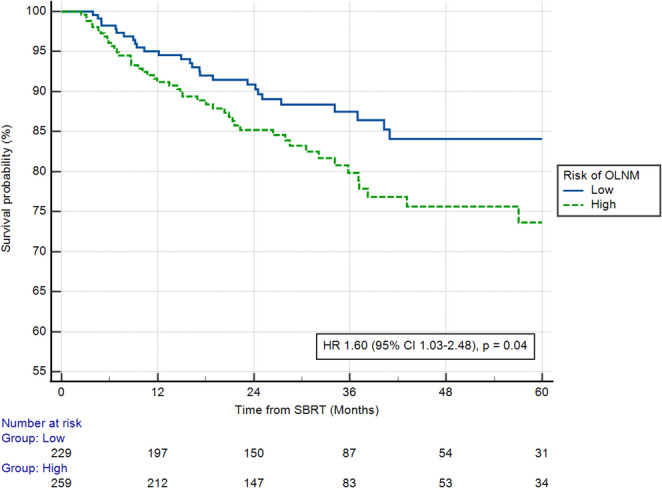
Regional relapse-free survival according to the ModelPET for the prediction of OLNM

With a median follow-up of 29.1 months, regional relapse occurred in 80 cases (16.39%). The median RRFS was not reached. The Model_PET_ was prognostic of RRFS as mean 5y-RRFS was significantly shorter in the high-risk group when compared to the low risk (ΔRRFS = 3.5 months, 95% CI 0.1–6.8, *p* = 0.04). Median RRFS was significantly shorter in the high-risk population when considering the whole Cohort C (HR 1.60, 95% CI 1.03–2.48, *p* = 0.04, Fig. 3). When differentiating the two subpopulations within the Cohort C, Model_PET_ was significantly associated with RRFS in the CHU Liège population but not in the CHU Brest population (Supplementary Figs. 3a and 3b, respectively). Regarding OS and applying the 12.5% probability threshold, the Model_PET_ was also prognostic of OS as mean 5y-OS was significantly shorter in the high-risk cohort (ΔOS = 3.6 months, 95% CI 0.04–7.3, *p* < 0.05). However, no statistically significant difference was seen for median OS (HR 1.13, 95% CI 0.90–1.42, *p* = 0.28, Supplementary Fig. 4).

Results on the sub-cohort of patients without histology (87 patients) were similar for RRFS with an HR of 1.86 (95% CI 0.60–5.78). Regarding OS, the Model_PET_ resulted in an HR of 0.97 (95% CI 0.57–1.67).

The Model_Combined_ was associated with none of the survival endpoints (*p* > 0.05).

### Focus on N2 prediction

N2 status was found in 4.5% of Cohort A and 7.1% of Cohort B. When refining the prediction for N2, the probability threshold rose to 14%. In the Cohorts A and B, the Model_PET_ respectively achieved a Bacc of 66.1/73.6%, Se of 55.6/87.5% and Sp of 76.6/59.6%.

**Fig. 4:**
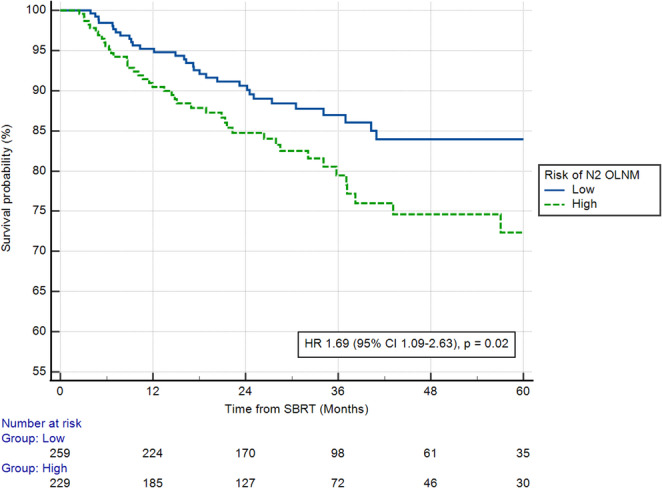
Regional relapse-free survival according to the ModelPET for the prediction of N2 status on the SBRT cohort

In the SBRT cohort, applying the 14% probability threshold, the Model_PET_ was associated with a significant impact on 5y-restricted mean RRFS (*p* = 0.03), median RRFS (HR 1.69 95% CI 1.09–2.63, *p* = 0.02, Fig. 4), 5y-restricted mean OS (*p* = 0.003) and median OS (HR 1.28 95% CI 1.02–1.60, *p* = 0.03, Supplementary Fig. 5).

### Radiomics quality score

The Radiomics Quality Score was 20/36 (Supplementary Table 4). Apart from the highlighted strengths (solid ground-truth, use of an external validation cohort and an application cohort, radiomics methodology in accordance with guidelines: IBSI compliant extraction of features, NeuroCombat harmonization and multi-reader segmentations, clear statistical report including decision-curve analysis), the main RQS shortfalls are the lack of phantom studies, the absence of inter/intra-observer segmentation robustness analysis, the retrospective design and the absence of cost-effectiveness analysis beyond DCA. Regarding the code availability, the MLP structure can be shared upon request while we already provide the ranking of each included features.

## Discussion

The findings of this study underscore the potential of radiomics-based predictive modeling in detecting OLNM among patients with localized NSCLC. The developed model demonstrated robust predictive performance across both surgical and SBRT cohorts, achieving a clinically meaningful high NPV. Notably, its prognostic value in the SBRT-treated cohort suggests that radiomics could play a role in refining patient stratification and treatment decisions. Given the challenges associated with OLNM detection—particularly in non-surgical settings—this model offers a non-invasive approach that could complement current imaging techniques and enhance clinical decision-making.

The study included three distinct cohorts of patients with localized NSCLC, each with specific characteristics. Namely, Cohort C had the oldest median age and a higher rate of squamous cell carcinoma. Across all cohorts, tumor stages varied, with the majority of cases classified as stage IA or IB, though Cohort B included some higher-stage cases (IIB–IIIA). Of note, the model was trained on a recent cohort (2010–2024) and validated on an older (1990–1996) surgical cohort including up to stage IIIA, while training was I–II. This design brings heterogeneity and enables a comprehensive evaluation of the radiomics model across different clinical settings.

Recent studies have explored various models for predicting OLNM in NSCLC patients. For example, Li et al. developed a nomogram incorporating clinical and imaging features to predict OLNM, demonstrating good calibration and discrimination [34]. Reaching an AUC of 0.85, the model combined clinical and biological features such as CA125 and CA153 levels that are not performed in routine clinical practice. Moreover, this study is limited by the lack of external validation. A systematic review by Li et al. assessed the diagnostic accuracy of CT and PET/CT radiomics in predicting lymph node metastasis, highlighting the potential of radiomics but also the need for further validation [16]. Out of the 22 included studies, only 7 included external validation. PET/CT radiomics appeared to be superior to CT radiomics AUC of 0.90 (95% CI: 0.87–0.93) vs. AUC 0.84 (95% CI: 0.79–0.88). This review did not differentiate training from validation cohorts. Zhong et al. published in 2023 the study with, to our knowledge, the largest validation cohort (1354 patients) [18]. Robust results were shown across the external cohort as well as a prospective cohort, with an NPV of 96.0% and 97.1% respectively. The higher PPV (48.3% and 58.6% should be highlighted when compared to our model (19.6%). However, the prospective cohort (999 patients) consists of 703 patients from the same center as the training cohort. While being different patients, this limits the interpretation of the model’s robustness to the variability of centers and PET/CTs. In our approach, Cohorts A and C were from largely different centers as only 52/488 patients from the Cohort C (10.7%) were from the same center as Cohort A. Cohorts A and B are two separate centers. When differentiating the two populations within Cohort C, Model_PET_ was significantly associated with RRFS in the CHU Liège population. The non-significant results in the CHU Brest population are difficultly interpretable given the small size of the cohort, the small number of events and the relatively high censoring.

We proposed an innovative approach developing a model on gold standard data, i.e. pathology samples and then evaluating the model on a separate external cohort treated with SBRT. A similar approach was previously tested [35] by Ni et al. who developed a model on a surgical cohort and validated the approach on patients treated with SBRT. Our analysis differs on several points. The rate of OLNM in our cohorts was closer to clinical practice (13% vs. 50% in the training cohort, 10.7% vs. 33% in the testing cohort). Our surgical testing cohort is a true external validation while the testing cohort used by Ni et al. was from the same center as the training cohort (temporal validation). The size of the SBRT cohort was greater (488 vs. 213 patients), with the majority of patients (89.3% vs. 76.1%) being from a center not included in the training of the model, thus providing a large external validation. We should also acknowledge the low rate of unknown histology (clinical diagnosis: 17.8% vs. 46% in the study by Ni et al.) as well as the inclusion of stage II NSCLC in our cohort (14.4%).

Inclusion of clinical features did not increase the performance of the prediction model when coming to external validation. While clinical features carry meaningful information, the Model_Combined_ did not hold its robustness on Cohort B possibly due to the differences in clinical parameters, especially age, between Cohorts A and B. Despite the global superiority of the Model_PET_ over the Model_Combined_ independently from the number of retained features, it’s also possible that the combination of a higher number of features per model led to a higher risk of overfitting, especially for a relatively rare event (OLNM rate of 10.7% in Cohort B).

We also provided results concerning N2 prediction. Interestingly, when applying the N1 probability threshold, the Model_PET_ was validated on both 5y-RRFS and 5y-OS but not on median OS. Only, when applying the N2 probability threshold, was the model prognostic of median OS. This confirms the possible dominant prognostic impact of N2 status on OS [1].

Our approach is particularly meaningful in light of the results of the SEISMIC study [10]. This study showed that EBUS had clinically significant changes in 18/124 patients. While 25% of patients were downstaged, 12% presented OLNM with insufficient dose coverage. Systematic EBUS could leverage the diagnostic fallouts of PET/CTs but its low availability limits its systematization. By providing an externally validated model on both surgical and SBRT cohorts with a negative predictive value of 96.7% (vs. 89.3% for the PET/CT), our model could be used on a daily basis as a tool for EBUS planning. A high NPV is the priority in such studies, to minimize the impact of false negatives. Patients with a cN0 PET/CT and a low calculated risk could move directly to surgery (or SBRT). For both surgical and radiotherapy patients, adapting the lymph node dissection template or the irradiation field seems not feasible, especially given the lack of localization capability for all OLNM prediction models, including ours. Patients at high risk (despite cN0 on PET/CT) should thus be offered a sampling of lymph node metastases (EBUS). Depending on the EBUS results (or solely on the model if not feasible), our model could also lead to adaptations in systemic therapies, all of which are subject to dedicated trials evaluating their benefit and toxicity. In surgical patients with a high OLNM risk, especially if N2 prediction, neoadjuvant/peri-operative immunotherapy could be considered based on the overall survival benefit in locally advanced NSCLC [36]. Furthermore, provided a dedicated trial, patients stratified at high-risk could benefit from concomitant systemic treatment such as immune checkpoint inhibitors. To our knowledge, apart from the I-SABR phase II study [37], the phase III trials (KEYNOTE-867 : NCT03924869 and PACIFC-4 : NCT03833154) have failed to demonstrate the superiority of adding ICI to lung SBRT when compared to SBRT alone for localized NSCLC. This highlights the need for better stratification tools.

The study’s limitations include its retrospective design, which may introduce selection bias. We acknowledge a dropout in performance between the training and the testing. This can be possibly explained by the difference in lymph-node dissection guidelines between the two cohorts (1990–1996 vs. 2010–2024) which may have brought heterogeneity regarding the minimum stations targeted at the time as well as the area that were dissected (mediastinal vs. hilar sampling). While gradient-based semi-automatic segmentation reduces the variability [38], our segmentation pipeline does not assess inter- and intra-reader consistency. The harmonization and normalization were performed on the three cohorts combining the methodologies developped by Fortin *et* al and Da-Ano et al., because of the heterogeneity of centers and temporal inclusion. Future work should assess other site-specific preprocessing plus fixed inter-center correction methodologies, to assess the impact of these pre-processing steps. The Model_PET_ was based solely on PET-derived radiomics features. While CT provides morphological data (location, infiltration, density), we couldn’t include it because contrast-enhancement was not standardized in the three cohorts, which would have led to an imbalance. Previous studies have shown robust results with CT-based models [35] and even higher when combining CT and PET [39] but lacked external validation, yet al.one in a cohort treated with radiotherapy. While Ni et al. were the first to provide such data with a CT-alone based model, we hereby provide results with a PET-alone based model and acknowledge the need to evaluate the value of a model combining data from both the CT and the PET. Furthermore, radiomic features can be abstract, making it challenging to interpret their biological significance. The first order mean feature was the most important feature. It approximates the mean tumour PET intensity, a feature that has been repeatedly associated with response to treatments in lung cancer [40]. Gray level Run Length Matrix is often associated with tumour aggressiveness/heterogeneity [22]. Providing an in-depth assessment of the molecular profile of tumours identified by the radiomic signature at high-risk of OLNM would be of interest. The choice of a MLP architecture was based on our previous works [27–30]. Nevertheless, given the global results of XGBoost approaches [41], comparing the two models is of clear interest. Finally, we have seen consistent results within the internal validation and external validation cohorts but after a drop from the training cohort, thus resulting in a moderate calibration in the validation cohorts. While already providing external validation, we believe that prospective and multi-center studies are needed to assess clinical implementability and benefit.

## Conclusion

This study presents a [^18^F]FDG PET radiomics-based predictive model for detecting OLNM in localized NSCLC, externally validated across both surgical and SBRT cohorts. The model demonstrated a high negative predictive value outperforming conventional PET/CT alone. Importantly, its prognostic significance in SBRT-treated patients suggests that radiomics could serve as a valuable tool for patient stratification and treatment optimization. By providing a non-invasive imaging-based triage tool to prioritize EBUS/mediastinoscopy in cN0 patients, this model has the potential to refine clinical decision-making. Prospective validation is warranted.

## Supplementary Information

Below is the link to the electronic supplementary material.

Supplementary Material 1 (DOCX 537 KB)

## Data Availability

Data from the Radiogenomics cohort: publicly available. Data from other cohorts: upon request to the corresponding author.

## Declarations

### Trial registration number

Liège: 2022/285, registered 8^th^ November 2022.

CHU Brest: 29BRC25.0162, registered 5^th^ June 2025.
