# Supplementary material for: Development and external validation of a FDG PET-based radiomics model predicting occult lymph node metastasis in non-small cell lung cancer patients
Source: Eur J Nucl Med Mol Imaging. 2026 Jan 16;53(6):3838–48. doi: 10.1007/s00259-025-07740-y (PMC13121205; doi:10.1007/s00259-025-07740-y)
Supplement: Supplementary file 1 — Supplementary Material 1 (DOCX 537 KB) [file 259_2025_7740_MOESM1_ESM.docx]

SUPPLEMENTARY MATERIALS

Supplementary Table 1: Description of all radiomics features retained after the feature set reduction step

Supplementary Table 2: Radiomics features combined in the Model_PET_, with respective importance

Supplementary Table 3: Calibration parameters for the Model_PET_ and the Model_Combined_ in the Cohorts A and B

Supplementary Table 4: Radiomics Quality Score

Supplementary Figure 1: Youden Index of all models according to the number of features in each separate sub-model

Supplementary Figure 2a-b: Calibration plots for the Model_PET_ and the Model_Combined_ in the Cohorts A (2a) and B (2b).

Supplementary Figure 3: Regional relapse free survival according to the Model_PET_ for the prediction of OLNM in the CHU Liège (3a) and CHU Brest (3b) populations

Supplementary Figure 4: Overall survival according to the Model_PET_ for the prediction of OLNM

Supplementary Figure 5: Overall survival according to the Model_PET_ for the prediction of N2 status

Supplementary Table 1: Description of all radiomics features retained after the feature set reduction step

| Description | Matrix | Filter | Radiomics Features |
| --- | --- | --- | --- |
| Wavelet-LHL_glcm_Autocorrelation | GLCM | Wavelet - LHL | Autocorrelation |
| Wavelet-LHL_glcm_Idmn | GLCM | Wavelet - LHL | Idmn |
| Wavelet-LHH_glrlm_HighGrayLevelRunEmphasis | GLRLM | Wavelet - LHH | High Gray Level Run Emphasis |
| Wavelet-HLH_glrlm_ShortRunEmphasis | GLRLM | Wavelet - HLH | Short RunEmphasis |
| Wavelet-HLH_glszm_ZonePercentage | GLSZM | Wavelet - HLH | Zone Percentage |
| Wavelet-HHL_firstorder_Variance | First order | Wavelet - HHL | Variance |
| Wavelet-HHL_glrlm_LongRunHighGrayLevelEmphasis | GLRLM | Wavelet - HHL | Long Run High Gray Level Emphasis |
| Wavelet-HHL_glszm_ZoneVariance | GLSZM | Wavelet - HHL | Zone Variance |
| Wavelet-HHH_glcm_JointEnergy | GLCM | Wavelet - HHH | Joint Energy |
| Wavelet-HHH_glrlm_ShortRunEmphasis | GLRLM | Wavelet - HHH | Short RunEmphasis |
| Wavelet-LLL_firstorder_Entropy | First order | Wavelet - LLL | Entropy |
| Wavelet-LLL_firstorder_InterquartileRange | First order | Wavelet - LLL | Interquartile Range |
| Wavelet-LLL_firstorder_Mean | First order | Wavelet - LLL | Mean |

Abbreviations: GLCM = Gray Level Co-Occurrence Matrix, GLRLM = Gray Level Run Length Matrix, GLSZM = Gray Level Size Zone Matrix, Wavelet filters: H = High, L = Low

Supplementary Table 2: Features combined in the Model_PET_ and the Model_Combined_, with respective importance

| Radiomics features | Model_PET_ | Model_Combined_ |
| --- | --- | --- |
| Wavelet-LHL_glcm_Autocorrelation | 2.2% | 4.4% |
| Wavelet-LHL_glcm_Idmn | 9.2% | 6.2% |
| Wavelet-LHH_glrlm_HighGrayLevelRunEmphasis | 11.6% | 5.5% |
| Wavelet-HLH_glrlm_ShortRunEmphasis | 5.8% | 6.4% |
| Wavelet-HLH_glszm_ZonePercentage | - | 6.8% |
| Wavelet-HHL_firstorder_Variance | 5.9% | 5.3% |
| Wavelet-HHL_glrlm_LongRunHighGrayLevelEmphasis | 13.0% | 6.3% |
| Wavelet-HHL_glszm_ZoneVariance | 12.3% | 7.5% |
| Wavelet-HHH_glcm_JointEnergy | - | 7.6% |
| Wavelet-HHH_glrlm_ShortRunEmphasis | 13.3% | 5.4% |
| Wavelet-LLL_firstorder_Entropy | 4.8% | 6.1% |
| Wavelet-LLL_firstorder_InterquartileRange | 5.5% | 6.0% |
| Wavelet-LLL_firstorder_Mean | 16.4% | 8.0% |
| Age | - | 17.5% |
| Histology | - | 4.2% |
| Gender | - | 1.4% |
| AJCC stage | - | 5.4% |

Abbreviations: Wavelet filters: H = High, L = Low, AJCC: American Joint Committee on Cancer (Lung classification, 9^th^ edition)

Supplementary Table 3: Calibration parameters for the Model_PET_ and the Model_Combined_ in the Cohorts A and B

| Cohort | Model | Intercept (α) | Slope (β) |
| --- | --- | --- | --- |
| Cohort A | Model_PET_ | +3.983 | 3.125 |
|  | Model_Combined_ | +1.429 | 1.792 |
| Cohort B | Model_PET_ | +0.404 | 1.487 |
|  | Model_Combined_ | –7.090 | –2.728 |

Supplementary Table 4: Radiomics Quality Score according to Lambin *et al*., Nat Rev Clin Oncol 2017

| Item # | RQS Criteria (Lambin et al. 2017) | Max points | Points awarded | Evidence / pointer (section, figure) |
| --- | --- | --- | --- | --- |
| 1 | Image protocol quality (documented protocols and/or use of public protocols) | 2 | 1 | Methods: PET/CT acquisition & preprocessing (Materials and Methods); PET-only rationale stated. |
| 2 | Multiple segmentations (robustness to segmentation variability) | 1 | 0 | Methods: single segmenter per cohort with RO review; no reproducibility experiment → 0. |
| 3 | Phantom study on all scanners (inter-scanner/vendor variability) | 1 | 0 | Not performed. |
| 4 | Imaging at multiple time points (test–retest / temporal variability) | 1 | 0 | Not performed. |
| 5 | Feature reduction or adjustment for multiple testing | 3 | 3 | Methods: univariate filtering (Mann–Whitney), Spearman de-correlation, reduced feature set for MLP. |
| 6 | Multivariable analysis with non‑radiomics features | 1 | 1 | Methods/Results: ModelCombined includes clinical variables (exploratory). |
| 7 | Detect and discuss biological correlates / interpretability | 1 | 1 | Discussion: qualitative interpretation of top PET features; Supplementary Table 2 (feature importances). |
| 8 | Cut‑off analyses / continuous risk reporting | 1 | 1 | Methods & Results: pre‑specified 12.5% risk cut‑off (Youden) locked for B/C; 14% for N2 exploratory. |
| 9 | Discrimination statistics with uncertainty; resampling | 2 | 2 | Results/Table 2: AUC (c‑statistic) with 95% CI; internal CV described. |
| 10 | Calibration statistics / plots (with uncertainty/resampling) | 2 | 2 | Calibration plots reported (Supplementary Figures 2a-b) |
| 11 | Prospective study registered in a trial database | 7 | 0 | Retrospective design; no preregistration. |
| 12 | Validation without retraining or cut‑off adaptation | 5 | 4 | External validation on multi‑institutional TCIA surgical cohort (Centers 2 & 3); 12.5% cut‑off applied unchanged. |
| 13 | Comparison to ‘gold standard’ / standard-of-care baseline | 2 | 2 | Text & Supplement: comparison vs PET-alone baseline; Decision Curve includes PET-alone (Supp. Fig. 2). |
| 14 | Potential clinical utility (e.g., decision‑curve analysis) | 2 | 2 | Supplementary Figure 2: DCA for training/testing; clinical triage narrative in Discussion. |
| 15 | Cost‑effectiveness analysis | 1 | 0 | Not performed. |
| 16 | Open science and data (scans/ROIs/code/features open) | 4 | 1 | External set B uses public TCIA scans (+1). No public ROIs/code/features export yet (0 for others). |
|  | TOTAL | 36 | 20 | Reported total in manuscript: 20/36 |

Supplementary Figure 1: Youden Index of all models according to the number of features in each separate sub-model


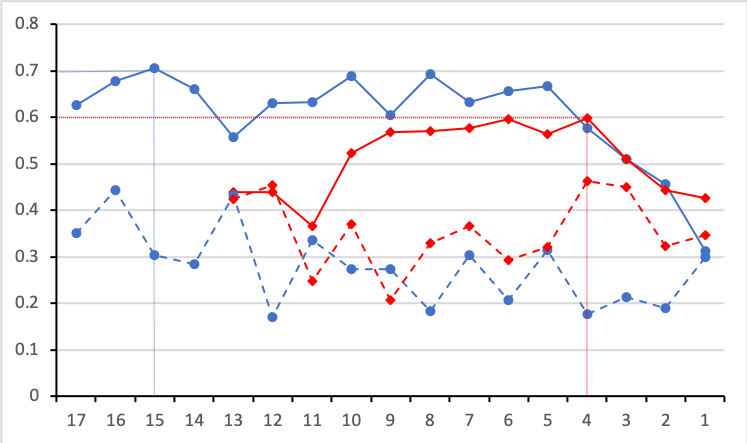


Caption: red: radiomics, blue: combined, full line: training cohort, dash line: testing cohort

Supplementary Figure 2a-b: Calibration plots for the Model_PET_ and the Model_Combined_ in the Cohorts A (2a) and B (2b).

2a: Calibration plots for the Model_PET_ and the Model_Combined_ in the Cohort A


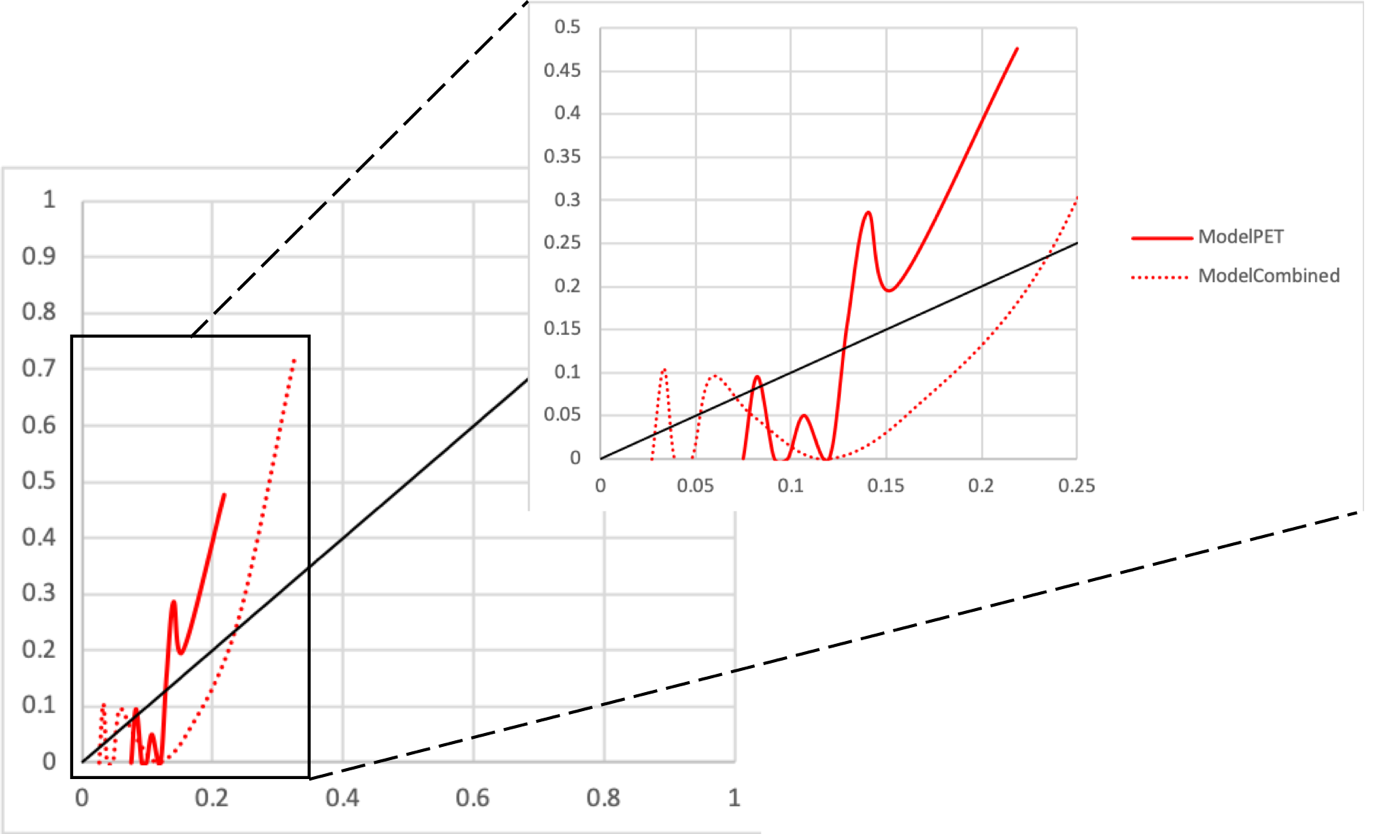


2b: Calibration plots for the Model_PET_ and the Model_Combined_ in the Cohort B


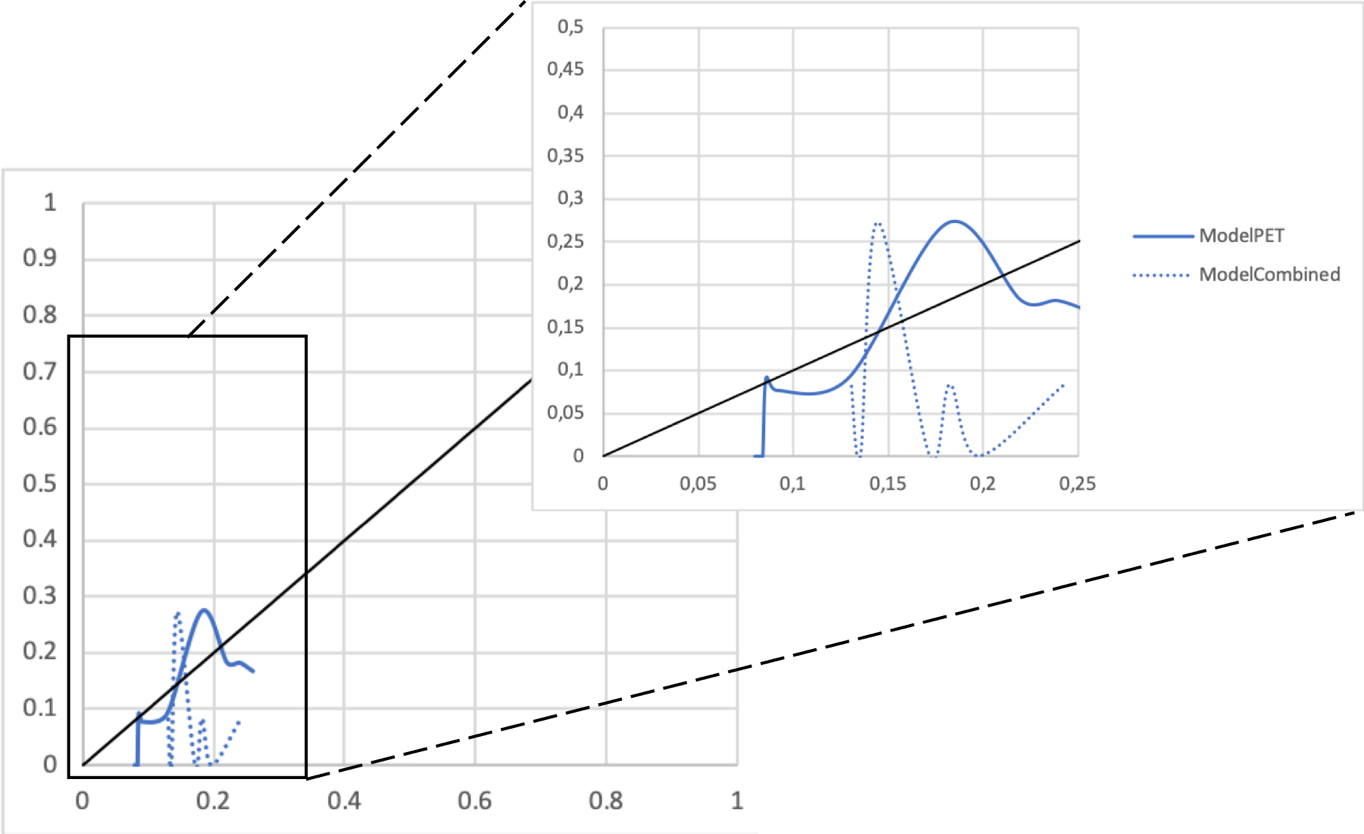


Supplementary Figure 3: Regional relapse free survival according to the Model_PET_ for the prediction of OLNM in the CHU Liège (3a) and CHU Brest (3b) populations

3a: Regional relapse free survival according to the Model_PET_ for the prediction of OLNM in the CHU Liège population


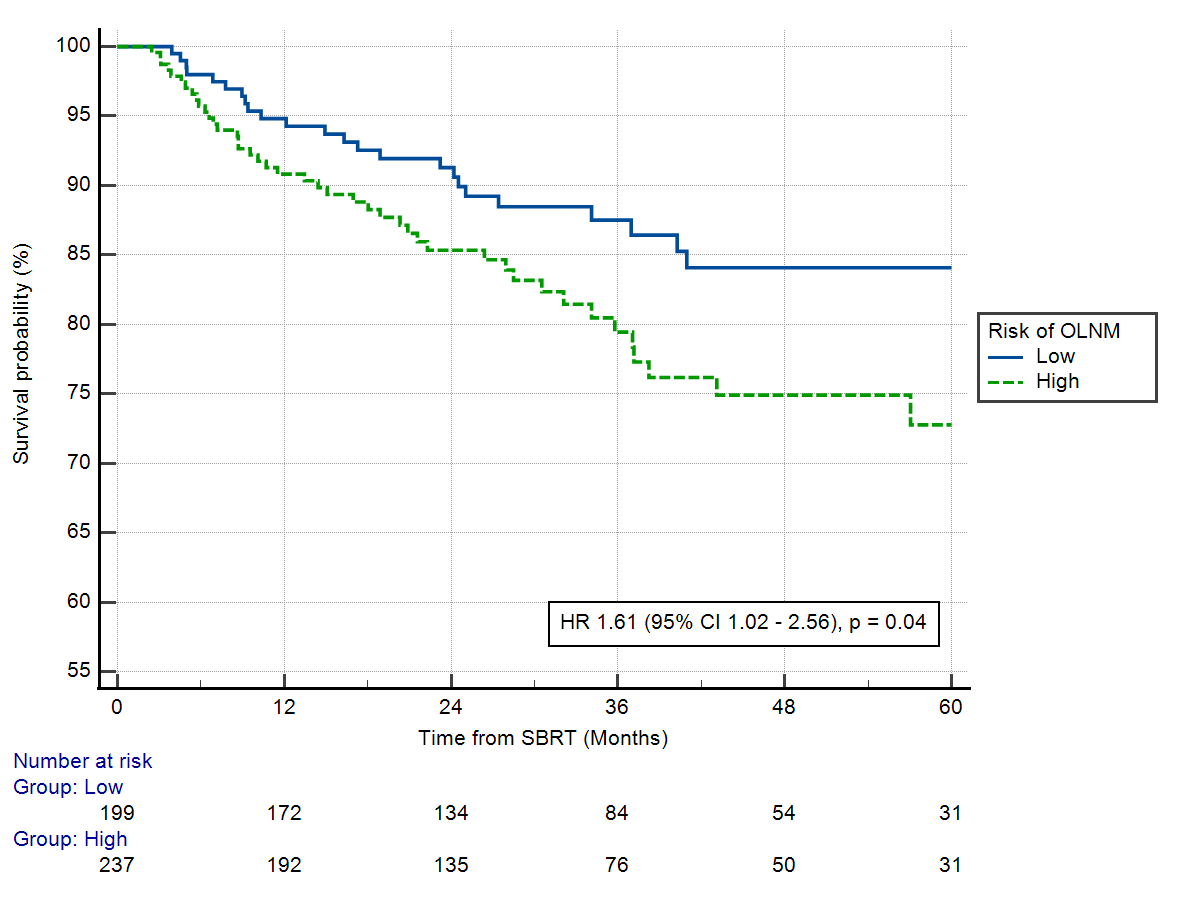


Abbreviations: OLNM = Occult Lymph Node Metastasis, Green: patients at high risk of OLNM based on the Model_PET,_ Blue: patients at low risk of OLNM based on the Model_PET_, HR: Hazard Ratio, CI= Confidence Interval

3b: Regional relapse free survival according to the Model_PET_ for the prediction of OLNM in the CHU Liège population


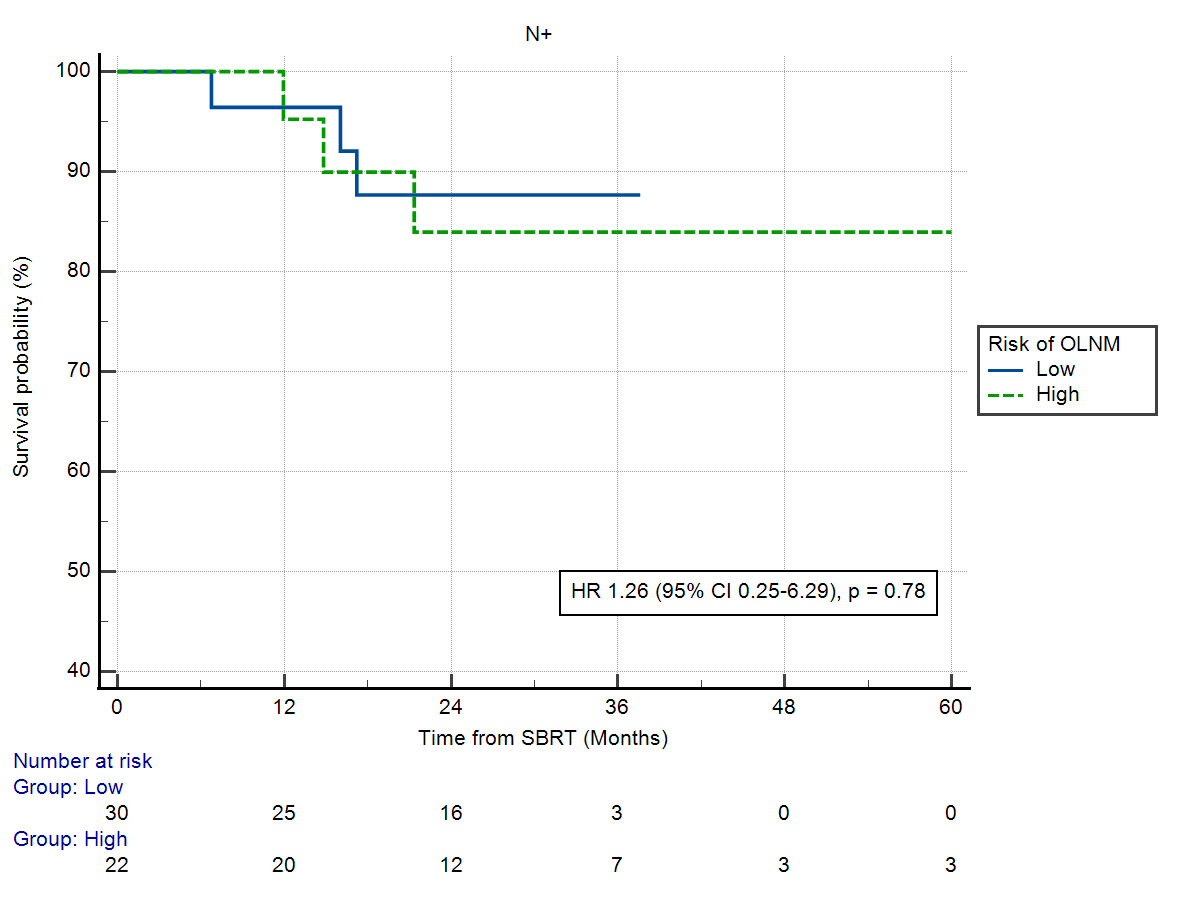


Abbreviations: OLNM = Occult Lymph Node Metastasis, Green: patients at high risk of OLNM based on the Model_PET,_ Blue: patients at low risk of OLNM based on the Model_PET_, HR: Hazard Ratio, CI= Confidence Interval

Supplementary Figure 4: Overall survival according to the Model_PET_ for the prediction of OLNM


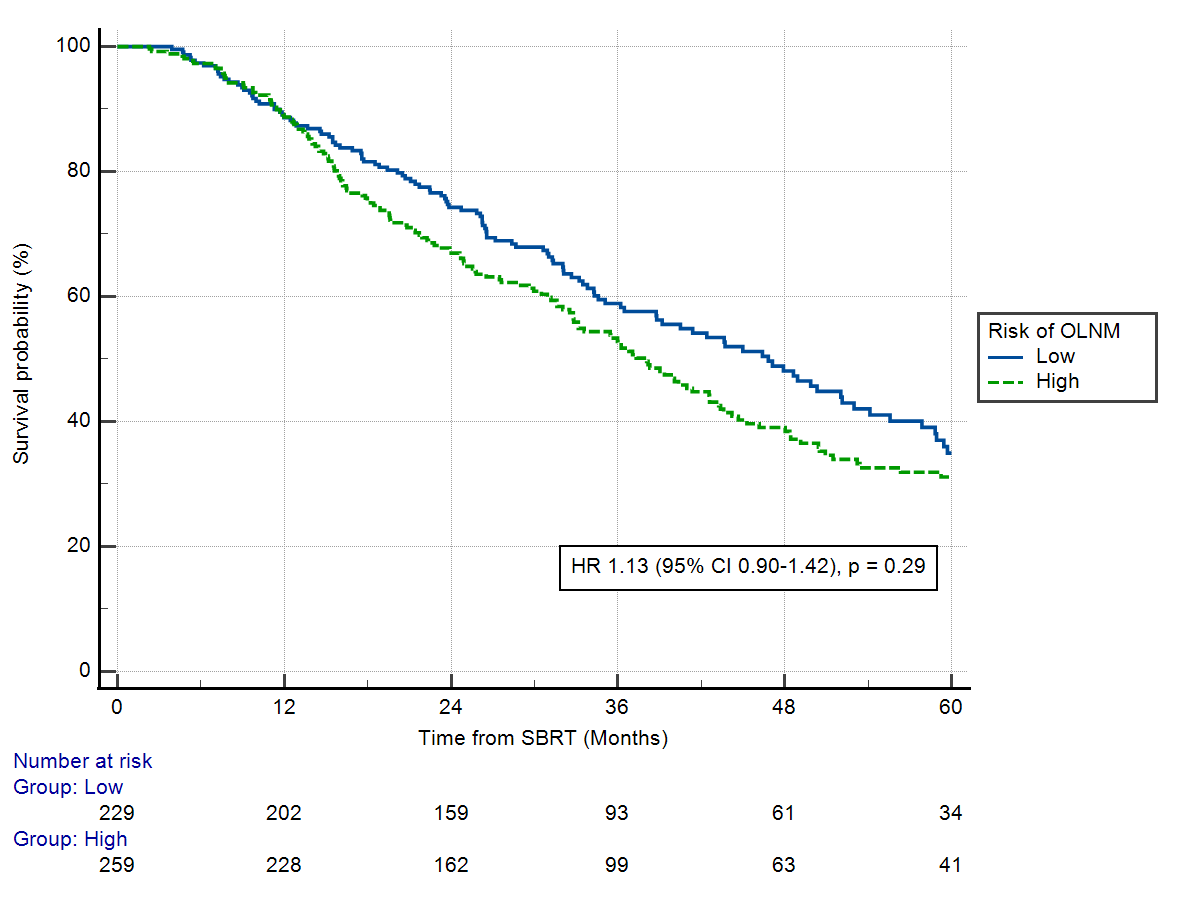


Abbreviations: OLNM = Occult Lymph Node Metastasis, Green: patients at high risk of OLNM based on the Model_PET,_ Blue: patients at low risk of OLNM based on the Model_PET_, HR: Hazard Ratio, CI= Confidence Interval

Supplementary Figure 5: Overall survival according to the Model_PET_ for the prediction of N2 status


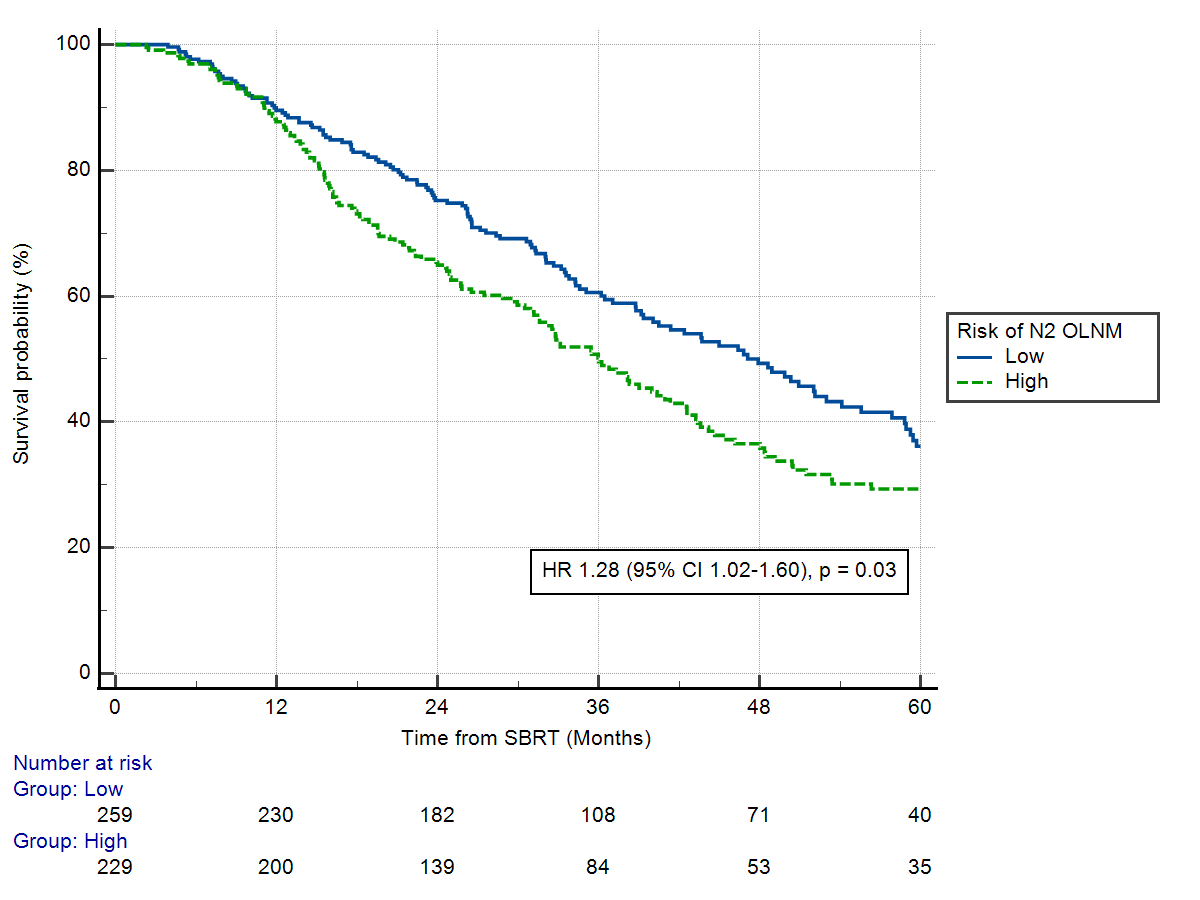


Abbreviations: OLNM = Occult Lymph Node Metastasis, Green: patients at high risk of OLNM based on the Model_PET,_ Blue: patients at low risk of OLNM based on the Model_PET_, HR: Hazard Ratio, CI= Confidence Interval
